# How do eubacterial organisms manage aggregation-prone proteome?

**DOI:** 10.12688/f1000research.4307.1

**Published:** 2014-06-27

**Authors:** Rishi Das Roy, Manju Bhardwaj, Vasudha Bhatnagar, Kausik Chakraborty, Debasis Dash

**Affiliations:** 1 GNR Knowledge Centre for Genome Informatics, Institute of Genomics and Integrative Biology, Council of Scientific and Industrial Research, Delhi, 110007, India; 2 Department of Computer Science, Maitreyi College, Chanakyapuri, Delhi, 110021, India; 3 Department of Computer Science, Faculty of Mathematical Sciences, University of Delhi, Delhi, 110007, India; 4 Department of Biotechnology, University of Pune, Pune, 411007, India

## Abstract

Eubacterial genomes vary considerably in their nucleotide composition. The percentage of genetic material constituted by guanosine and cytosine (GC) nucleotides ranges from 20% to 70%.  It has been posited that GC-poor organisms are more dependent on protein folding machinery. Previous studies have ascribed this to the accumulation of mildly deleterious mutations in these organisms due to population bottlenecks. This phenomenon has been supported by protein folding simulations, which showed that proteins encoded by GC-poor organisms are more prone to aggregation than proteins encoded by GC-rich organisms. To test this proposition using a genome-wide approach, we classified different eubacterial proteomes in terms of their aggregation propensity and chaperone-dependence using multiple machine learning models. In contrast to the expected decrease in protein aggregation with an increase in GC richness, we found that the aggregation propensity of proteomes increases with GC content. A similar and even more significant correlation was obtained with the GroEL-dependence of proteomes: GC-poor proteomes have evolved to be less dependent on GroEL than GC-rich proteomes. We thus propose that a decrease in eubacterial GC content may have been selected in organisms facing proteostasis problems.

## Introduction

Eubacterial organisms have genomes that vary largely in their nucleotide compositions. In this kingdom, the GC content varies from 20% to 70% of the genome and this large variation has been documented in a number of reports that have aimed to explain it ^1– 3^. The amino acid compositions are also different in eubacterial proteomes due to the variation of GC content ^4^. It has been reported that these difference of amino acid compositions alter the characteristics of proteomes and as a consequence, proteins of GC-poor genomes are more prone to misfolding and aggregation compare to GC-rich genomes ^5, 6^. It has been hypothesized that GroEL plays a major role, if not an essential role, in the evolution of GC-poor organisms by buffering deleterious mutations that are fixed due to population bottlenecks ^7– 9^. This has been supported by the observation that many of the small GC-poor endosymbionts tend to overexpress GroEL ^10– 12^.

However, the proposed chaperone dependence of GC-poor organisms does not explain why some of the GC-poor endosymbionts of the mycoplasma group have lost the *groEL* copy from their genome ^13^. It is notable that these are the only known eubacterial organisms to have lost this gene. This observation led us to test the proposed relationship of GC poorness of genome with the aggregation propensity of the encoded proteome.

Obtaining information on the aggregation propensity of proteins from different organisms is a challenging task. However, there has already been a careful characterization of the aggregation propensity of different *Escherichia coli* proteins that was conducted in a high-throughput manner ^14– 16^. Kerner *et al.* classified the GroEL substrates into Class I, II or III based on the interaction strength and on the stringency of their requirement for GroEL. Class III (C3) substrates were completely dependent on GroEL for folding, whereas Class II (C2) substrates were partially dependent. Class I (C1) proteins interacted weakly with GroEL and were able to fold spontaneously. In a trivial approach, homologs of GroEL-dependent proteins may be identified in other organisms ^13, 17^. This approach however fails to predict the evolution of protein dependence on GroEL correctly, as the sequence differences between species have the potential to introduce or remove kinetic traps from folding pathways, thereby altering their dependence on GroEL. In addition to the solubility of the *E. coli* proteome in a chaperone-free system, substrates of another chaperone DnaK were also identified by two independent research groups ^18, 19^. Applications developed primarily on machine learning algorithms to classify soluble or GroEL substrates ^16, 18, 20– 24^ are already available. However, these classifiers have not been trained with curated data prepared from multiple experimental results ^14, 15, 18, 19^. In this study, we have constructed a more reliable training dataset to build classifiers to determine the aggregation propensity and GroEL dependency in 1132 eubacterial proteomes, based solely on the amino acid sequences. We show a distinct trend in the aggregation propensity of proteins of an organism in relation to the GC content. Surprisingly, aggregation propensity decreased with lower GC content independent of symbiotic characteristics, suggesting that GC-poor organisms have indeed evolved a proteome that is devoid of aggregation-prone proteins.

## Materials and methods

### Data source

**Figure 1.**
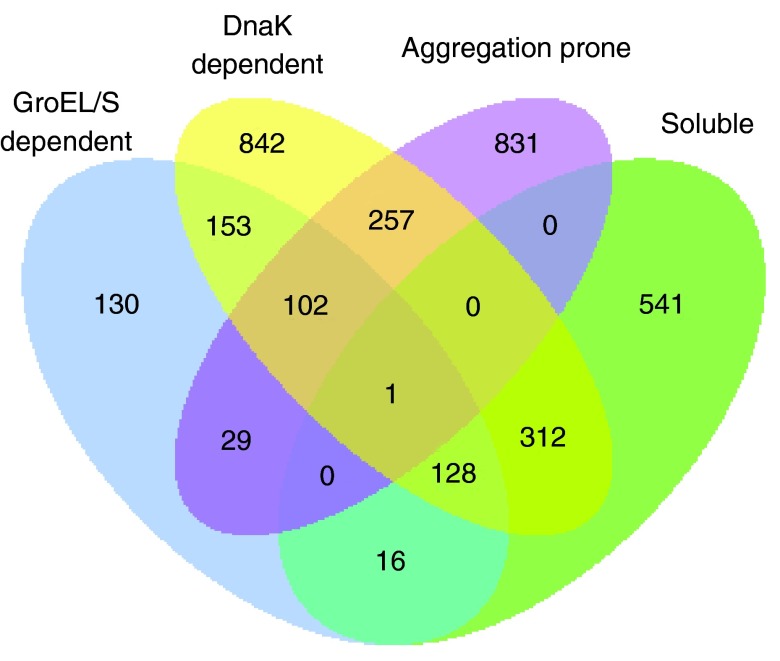
Integration of independent studies. A Venn diagram of proteins of *E. coli* identified by different experimental studies shows that ~45% of soluble proteins reported by Niwa *et al.* overlap with GroEL/S or DnaK substrates (soluble proteins are defined as having solubility >70% and aggregation-prone proteins have solubility <30%).

The aggregation-prone proteins of the eSOL database ^18, 25^ are dependent on the chaperone network of *E. coli* to get their three dimensional native structure. GroEL and DnaK are two important components of this network and their substrates have been extensively studied via different experimental methods ^14, 15, 19, 26^. The integration of all the available information reveals that about half (457) of the soluble or chaperone-independent proteins identified by Niwa *et al.* were found to be GroEL- or DnaK-dependent ^18^ ( Figure 1). To construct a more reliable training set, we removed these proteins from the soluble set. Thus, proteins identified as chaperone-dependent by more than one study, were only considered as aggregation-prone proteins. Furthermore, the proteins which were more than 30% (amino acid) sequence similarity among the remaining proteins were removed using CD-HIT ^27^ clustering program. Therefore the final training set comprised of 502 aggregation prone and 475 soluble proteins.

### Classifier building

The classifiers in this study were built with Pro-Gyan ^28^ software. Pro-Gyan builds classifiers directly from training data set given in FASTA format by selecting relevant features from a large number of unbiased features. Following metrics which are useful to evaluate performance of machine learning classifiers were reported by Pro-Gyan.

**Accuracy(Acc)= (TP + TN)/(TP + TN + FP + FN)**

**Sensitivity or Recall (Sn) = TP/(TP + FN)**

**Sencificity (Sp) = TN/(FP + TN)**

Matthews correlation coefficient (MCC) = (TP*TN–FP*FN)/ $\sqrt{\left\{(TP+FP)*(TN+FN)*(TP+FN)*(TN+FP)\right\}}$

where TP = True Positive, TN = True Negative, FP = False Positive, FN = False Negative predicted by the classifier.

Additionally, receiver operating characteristic (ROC) curves and area under this curve (AUC) ^29^ were also generated.

### Analysis on microbial genomes

The protein sequences of microbial genomes were downloaded from the Microbial Genome Database ^30^ (archive no. mbgd_2011-01). To identify the chaperonins in the microbial organisms, chaperonin homologs were searched for using BLAST (e-value 1*10-4) against a chaperonin database cpnDB ^31^ downloaded on June 2011. The 16S rRNA nucleotide sequence of *E. coli* was acquired from SILVA ^32^ and homologous were searched for in other microbial organisms using BLAST (e-value 1*10-4). GC contents for microbial genomes were calculated using following equation

GC content = (G + C)/(total bases),

where G = number of guanosine and C = number of cytosine.

### Statistical analysis

The Kendall correlation and analysis of covariance were performed in R ^33^ statistical computing environment using the package ‘stats’ version 2.15.3. To account the effect of evolution on different traits of bacterial genomes, we performed phylogenetic independent contrast through the PDAP ^34^ module on Mesquite ^35^ application.

## Results and discussion

### Development of machine learning tool to identify aggregation-prone proteins

**Figure 2.**
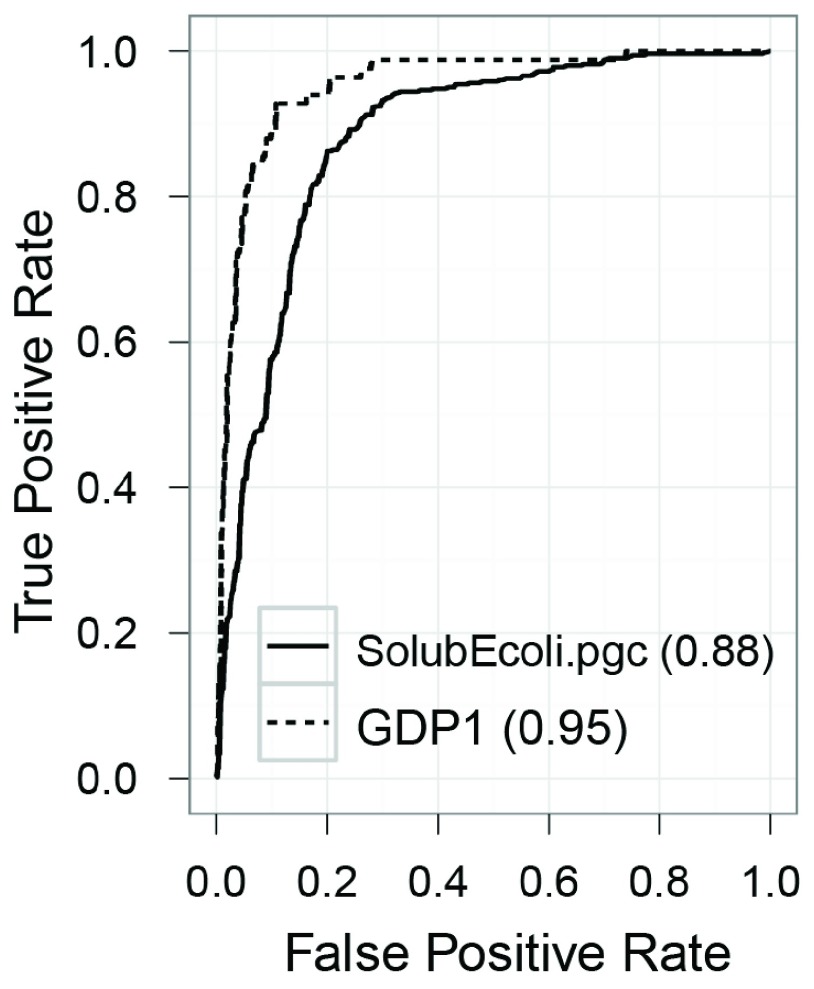
Receiver operating characteristic (ROC) curves. ROC curves of the soluble protein classifier (SolubEcoli.pgc) and the GroEL obligate protein classifier (GDP1.pgc). The areas under the curves (AUC) are given in the legend.

Recently protein solubility has been carefully measured in a chaperone-free system and the information has been made available through the eSol database ^18^. Few classification models developed on this database can segregate soluble proteins from chaperone-dependent proteins ^22– 24^. However, these web-based classifiers are not suitable to classify large numbers of proteomes, and their soluble or negative training dataset (proteins not aggregation-prone or soluble) are not carefully curated, as most of the soluble proteins from eSol database are substrates of DnaK ^19^ or GroEL ^14, 15^ ( Figure 1). Therefore we built a classifier containing a curated list of aggregation-prone proteins and soluble proteins. The classifier was built using Pro-Gyan ^28^ which generates 5038 different features from a set of class labelled protein sequences and selects the “maximum relevant minimum redundant” feature subset. Finally, the tool built a support vector machine (SVM) ^36^ classifier by five-fold cross validation. The classifier attained an accuracy of 83.21% with 0.66 MCC ( Table 1). Although Pro-Gyan generated classifier was trained with a rigorously curated training data set, it performs equivalent to Fang *et al.*’s classifier and better than others ^22– 24^. The receiver operating characteristic (ROC) curves of the classifier are shown in Figure 2. For interested users, the classifier is available in ZENODO ( https://zenodo.org/record/10442).

**Table 1.** Comparison of previous classifiers with our classifier.

Method	Sensitivity	Specificity	Accuracy	AUC	MCC
SVM ^25^			80		
J48 (decision tree algorithm) ^23^			72	0.72	
VTJ48 (visually tuned J48) ^23^			76	0.81	
Fang *et al.* ^22^	82.00	85.00	84	0.91	0.67
SolubEcoli.pgc*	**86.25**	**80.00**	**83.21**	**0.88**	**0.66**

* Built on a curated training data set.

### Discriminating features of aggregation prone proteins

To build the classifier, Pro-Gyan ^28^ selected 24 relevant features through an automated process. The top ten significant (by Mann-Whitney test) features were the sequence patterns, the pseudo amino acid composition ^37^ of phenylalanine (F), aspartic (D) and glutamic (E) acid, the distribution of positively charged amino acids, the features calculated from FoldIndex ^38^ and the auto-correlation of hydrophobicity and relative mutability ( Table 2). The remaining selected features ( Table 2) were enriched with auto-correlation measurement of amino acid indices such as steric parameter, free energy, accessible surface area, polarizability, residue volume etc. The features which represent patterns of physico-chemical properties encrypted in protein sequences were unique to SolubEcoli.pgc when compared to earlier methods.

**Table 2.** Selected features of proteins used to build the “SolubEcoli.pgc” classifier.

Serial no.	Feature id†	Description	p-value*
1	SW_SOC2	Quasi-sequence-order calculated from physicochemical distance matrix ^50^.	2.20E-16
2	PPR	Distribution of positively charged amino acids in sequence pattern ^51^.	2.20E-16
3	H(8)M	Amino acid pair composition of histidine to methionine with 8 gaps ^52^.	2.33E-15
4	M-B(Hydr)1	Moreau-Broto auto correlation (lag 1) of amino acid index; hydrophobicity ^53^.	2.24E-08
5	PseAAC_T1_3	Pseudo amino acid composition of aspartic acid (D) ^37^.	9.45E-06
6	PseAAC_T1_5	Pseudo amino acid composition of phenylalanine acid (F) ^37^.	6.87E-05
7	FI_16_psavgl	Average length of folded segments of proteins according to FoldIndex ^38^.	8.14E-05
8	PseAAC_T1_4	Pseudo amino acid composition of glutamic acid (E) ^37^.	0.000542
9	Dstrbu_Pol_2:3	Distribution of amino acids according to polarizability ^54^.	0.001289
10	M-B(mutblty)6	Moreau-Broto auto correlation (lag 6) of amino acid index; relative mutability ^53^.	3.65E-03
11	T	Composition of amino acid Threonine ^53^.	5.00E-03
12	Mrn(vlum)27	Moran auto correlation (lag 27) of amino acid index; residue volume ^53^.	0.00926
13	Mrn(Polar)22	Moran auto correlation (lag 22) of amino acid index; polarizability ^53^.	0.013
14	M-B(mutblty)9	Moreau-Broto auto correlation (lag 9) of amino acid index; relative mutability ^53^.	0.01988
15	Geary(sterc)4	Geary auto correlation (lag 4) of amino acid index; steric parameter ^53^.	0.03536
16	M-B(mutblty)24	Moreau-Broto auto correlation (lag 24) of amino acid index; relative mutability ^53^.	0.05416
17	M-B(Hydr)12	Moreau-Broto auto correlation (lag 12) of amino acid index; hydrophobicity ^53^.	5.92E-02
18	Mrn(RsdAcc)24	Moran auto correlation (lag 24) of amino acid index; residue accessible surface area in tripeptide ^53^.	0.1077
19	Mrn(Hydr)23	Moran auto correlation (lag 23) of amino acid index; hydrophobicity ^53^.	0.2106
20	Geary(Free)13	Geary auto correlation (lag 13) of amino acid index; free energy ^53^.	0.3271
21	Comp_Vol_2	Composition of normalized van der Waals volume of amino acids of range 2.95–4.0 ^53^.	0.4631
22	Geary(vlum)20	Geary auto correlation (lag 20) of amino acid index; residue volume ^53^.	4.95E-01
23	Geary(Free)14	Geary auto correlation (lag 14) of amino acid index; free energy ^53^.	0.499
24	M-B(vlum)30	Moreau-Broto auto correlation (lag 30) of amino acid index; residue volume ^53^.	0.9559

†Internal feature id of the Pro-Gyan application.

### Genome wide prediction of aggregation prone proteins

**Figure 3.**
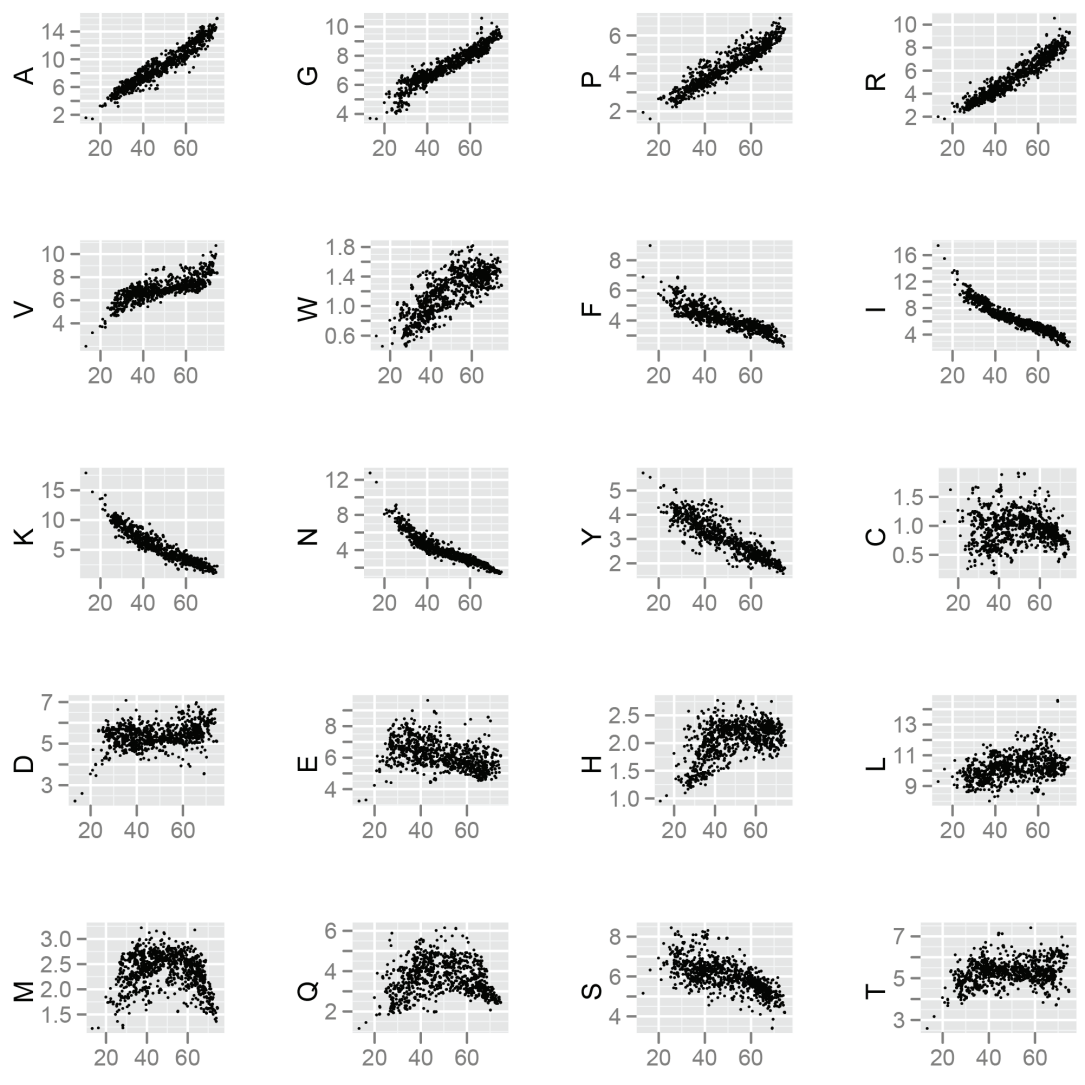
Composition of basic amino acids over ~1100 eubacterial genomes. The x-axis of each subplot shows for GC composition of each genome whereas y-axis shows corresponding amino acid composition.

**Figure 4.**
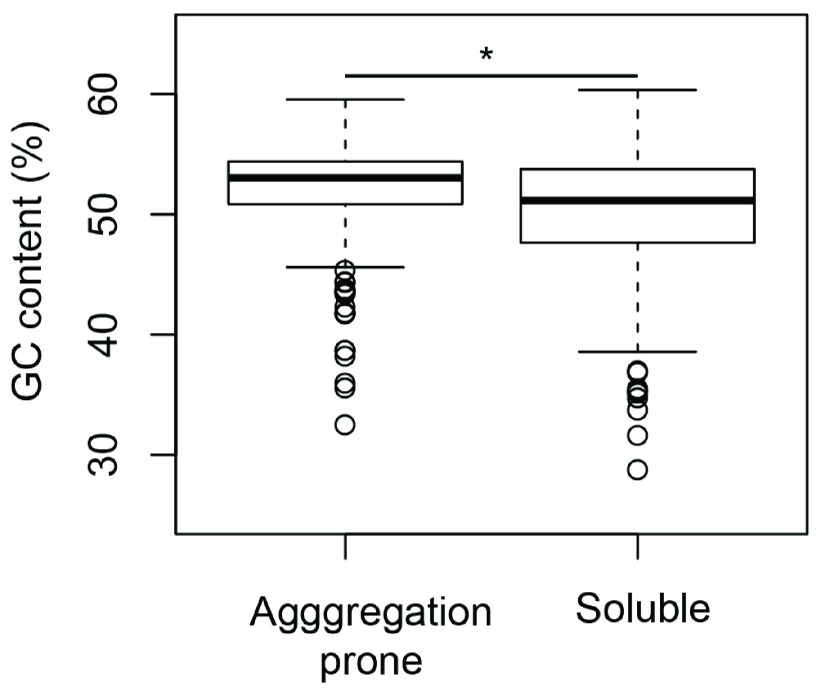
Aggregation-prone proteins are richer in GC-content than soluble proteins. In *E. coli*, aggregation-prone proteins contain higher GC-content than soluble proteins. Mann-Whitney test p-value (*) is 1.3e-15.

**Figure 5.**
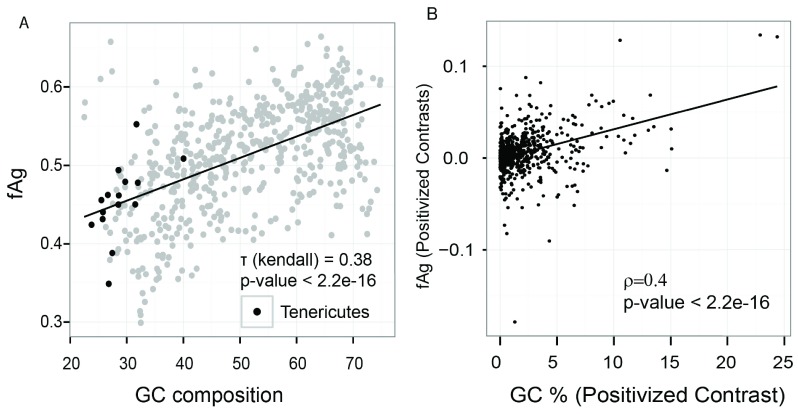
GC content is associated with fAg. ( **A**) GC content of the genome correlates with the fraction of proteome that is aggregation-prone (fAg) (analysis of 570 bacterial genomes using the classifier). Rank-based correlation is provided along with the p-value. The black line shows a linear regression model. ( **B**) The relationship between GC content and fAg was obtained through a phylogenetically independent contrast method (570 bacteria). A positive correlation (0.4) was identified between GC content and fAg (p-value < 2.1e-16).

From the analysis of features, it was noticed that the compositions of amino acids are significantly different within aggregation prone and soluble proteins. Sequence features of amino acids have been used to understand protein overexpression related to toxicity ^39^. Additionally, it has been also shown that the amino acid composition is drastically altered in organisms with GC-poor genomes ^4, 40^. There are multiple amino acids that change in frequency as a function of GC content ( Figure 3) and this change that has been attributed to the difference in the GC content in the codons of these amino acids. On the basis of these differences, it has been reported that proteins encoded by GC-poor organisms should be more prone to aggregation than proteins encoded by GC-rich organisms ^5, 6^. However, the GC composition of the training data showed that aggregation-prone proteins were significantly more GC-rich than the soluble proteins ( Figure 4, Mann-Whitney test p-value = 1.3e-15). Subsequently, we sought to verify the fraction of aggregation-prone proteins across different bacterial proteomes. We used the SolubEcoli.pgc classifier to predict aggregation-prone proteins in 1132 eubacterial species. Our prediction on bacterial genomes showed that the fAg (aggregation prone proteins as fraction of proteome) of a genome correlates positively with the GC composition (Kendall tau=0.38 p-value < 2.2e-16) ( Figure 5A). We further examined the correlation, with respect to phylogenetic ancestry, using the Mesquite system ^35^, because the Kendall correlation assumes that observations are independent even if organisms are linked through common ancestors ^41^. The required phylogenetic tree was constructed from the 16S rRNA gene sequences of 570 bacteria ^42^. We found a significant correlation (0.4, p-value < 2.2e-16) between independent contrasts of GC content and fAG ( Figure 5B). This corroborated well with the difference seen between soluble and aggregation-prone proteins in *E. coli* ( Figure 4). Thus the increase in the GC composition of a genome may encode proteome that harbours a higher fraction of aggregation-prone proteins.

**Figure 6.**
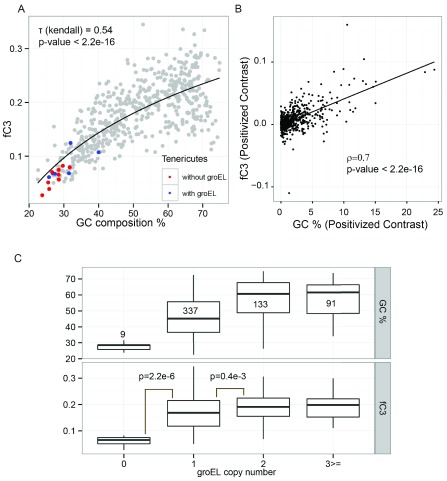
Decrease in GC content is associated with decrease in fC3. ( **A**) Correlation of GC content with the fraction of the proteome that is GroEL obligate (fC3) over 570 bacterial genomes. Members of the phylum Tenericutes with and without the *groEL* gene are coloured in blue and red, respectively. Rank-based correlation is provided along with p-value. The black line shows a logarithmic regression model. ( **B**) A positive correlation (0.7) was identified between independent contrast of GC content and fC3 with respect to phylogenetic information of bacterial genomes (570 bacteria, p-value < 2.2e-16). ( **C**) The organisms were classified based on the number of *groEL* genes present in the genome. fC3 exhibited a significant increase with an increase in the number of genome-encoded *groEL* copies. The p-values were calculated by Mann-Whitney test using two-sided hypothesis.

This is in contrast to previous reports hypothesizing that GC-poor organisms have unstable and aggregation prone proteomes. Notably, the earlier hypothesis that GC poorness is associated with GroEL-dependent aggregation-prone proteomes was based on the observation that GroEL is overexpressed in GC-poor organisms. Therefore, to segregate GroEL-dependent proteins from aggregation-prone proteomes, we developed another classifier (ZENODO, https://zenodo.org/record/10442/) trained with 475 curated soluble and 83 GroEL obligate (Class 3 or C3) proteins ^14^. The classifier achieved an accuracy of 92.29% with MCC of 0.69. We used GDP1.pgc to identify the C3 proteins within aggregation-prone proteins (predicted by SolubEcoli.pgc) to examine the evolution of the GroEL-dependent proteome with GC composition. Indeed we found that the fC3 (fraction of C3 proteins) of bacterial proteome are more correlated with GC content than the fAg fraction (Figure 6A). The phylogenetically independent contrasts of fC3 and GC also correlated strongly (0.7, p-value < 2.2e-16, Figure 6B). The phylum Tenericutes, members of which have GC-poor genomes, was predicted to encode less GroEL-dependent proteins. Mycoplasma and Ureaplasma are the main genera of the phylum Tenericutes and many species of these groups lack GroEL ^43^. In our analysis, we also observed that the Tenericutes without GroEL (red dots in Figure 6A) had very few fC3 proteins. This motivated us to investigate the effect of *groEL* copy number on misfolded proteins. Interestingly, there was a strong correlation between the *groEL* copy number and the fraction of genome coding for C3 proteins ( Figure 6C). Due to the presence of noise in the experimental data, we tried to benchmark the classifiers. Fujiwara *et al.* reported that five C3 homologs of *groEL*-lacking *Ureaplasma urealyticum* are soluble in GroEL depleted cells ^26^. Hence, we also examined the tolerance of our classifiers by predicting the GroEL dependency of these homologs. Four of these homologs were predicted to be GroEL independent with a high confidence score ( Table 3). Overall, the results indicated that C3 proteins and in general aggregation-prone proteins do decrease with the GC content of genomes.

**Table 3.** Evaluation of classifiers on five C3 homologous proteins of groEL-lacking *Ureaplasma urealyticum*. The homologous were found in *U. urealyticum* by NCBI BLAST at a threshold of E value of 1e45. Then the aggregation propensity and GroEL dependency of these proteins were classified by SolubEcoli.pgc and GDP1.pgc.

C3 homologous proteins in *Ureaplasma urealyticum*	E value	Accession	Is aggregation prone? (classifier: SolubEcoli.pgc)	Is GroEL dependent? (classifier: GDP1.pgc)
UuMetK	2e-99	YP_002284849.1	Yes (0.739)	No (0.804)
UuDeoA	2e-80	WP_004026878.1	Yes (0.586)	No (0.938)
UuCsdB	4e-62	D82890	Yes (0.884)	Yes (0.665)
UuGatY	8e-46	H82870	Yes (0.672)	No (0.973)
UuYcfH	7e-41	E82944	Yes (0.518)	No (0.881)

### Correlation of GC content with protein solubility is independent of the population bottleneck

**Figure 7.**
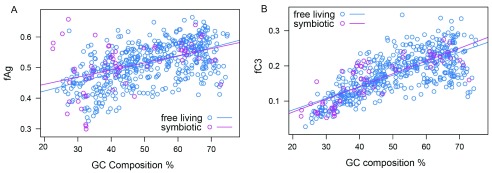
fAG and fC3 are correlated to the GC content independent from the species habitat. The ANCOVA test on 570 organisms showed that a symbiotic relationship has no significant effect or interaction with GC content on the aggregation propensity or GroEL-dependency of the proteins of an organism.

Endosymbionts are crucial to this study as the literature suggests that these organisms have undergone bottlenecks during evolution ^44^. It is hypothesized that these organisms have accumulated more deleterious mutations compared to non-endosymbionts ^8^. If this were true then endosymbionts should show a greater aggregation propensity or dependence on GroEL than that predicted by the GC content of free-living eubacterial species. To measure the impact of a symbiotic relationship on C3 proteins, we performed an analysis of covariance ANCOVA on 570 eubacterial species ^42^. There was no significant effect of a symbiotic relationship on fAG/fC3 (p-value 0.24/0.65, Data set) or significant interaction (p-value=0.36/0.38) with GC composition ( Figure 7). Thus we were unable to obtain proof for any association of a bottleneck in evolutionary history with protein aggregation propensity. Therefore we rule out the possibility of bottleneck evolution as the reason for the evolution of GroEL-independent proteomes like *Ureaplasma* and GroEL-independent mycoplasma species.

Application of SolubEcoli.pgc and GDP1.pgc classifiers Proteome wide prediction of GroEL obligatory protein fraction (fC3) and aggregation prone protein fraction (fAg) in 1132 eubacterial genomes with their genome size, GC content and GroEL copy number.

## Conclusions

Several machine learning (ML) classifiers have been developed to predict aggregation-prone or GroEL-dependent proteins, but very few of them used data sets generated and curated from multiple experimental studies. Our classifiers were based on curated data from multiple studies and performed well also against the false positive C3 homologs of *Ureaplasma*, showing accuracy and noise tolerance. According to previous theories, GC-poor organisms might have evolved through population bottlenecks. This allows mildly deleterious mutations to be fixed in the population with a high probability ^2, 44^. It has been hypothesized that the GC-poor genomes that accumulated a large number of deleterious mutations in the course of evolution, through population bottlenecks and hence harbour proteins that are aggregation-prone. Although overexpressions of chaperones are observed in GC-poor organisms that have reduced genomes, there are also other GC-poor organisms that lack GroEL. Our work provides strong evidence that the general stability of the proteome increases with the decrease in GC content of eubacterial genomes. Decrease in GC content restricts the amino acid space that the organism can sample, thereby compromising protein evolution. We hypothesise that, even with this limited amino acid space, GC-poor organisms are still abundant as growth is facilitated under conditions that compromise protein folding capacity. This antagonism between ability to evolve and folding advantage could be crucial in facilitating protein evolution in the presence of chaperones and other folding machineries ^45– 48^.

Our work suggests that organisms facing continuous proteostasis problems would tend to shift towards a more GC-poor genome. This is supported by findings of Xia *et al.* ^49^ who have reported that the preponderance of GC to AT conversions during high temperature laboratory adaptation of *Pasteurella multocida*. Further *in vitro* evolution experiments will be required to demonstrate whether laboratory adaptation to low GC content may provide folding advantage.

## Data availability

*F1000Research*: Dataset 1. Application of SolubEcoli.pgc and GDP1.pgc classifiers, 10.5256/f1000research.4307.d29624 ^55^.

ZENODO: Training data of protein classifier SolubEcoli.pgc and GDP1.pgc, doi: 10.5281/zenodo.10442 ^56^.
