# Supplementary material for: How do eubacterial organisms manage aggregation-prone proteome?
Source: F1000Res. 2014 Jun 27;3:137. [Version 1] doi: 10.12688/f1000research.4307.1 (PMC4193397; doi:10.12688/f1000research.4307.1)
Supplement: Peer review history

# Peer review: Referee response for version 1

**DOI:** 10.5256/f1000research.4611.r6283

**Published:** 2014-10-01

**Authors:** Annalisa Pastore

**Affiliations:** 1 MRC National Institute for Medical Research, London, UK

The genesis of this paper is the proposal that genomes containing a poor percentage of guanosine and cytosine (GC) nucleotide pairs lead to proteomes more prone to aggregation than those encoded by GC-rich genomes. As a consequence these organisms are also more dependent on the protein folding machinery. If true, this interesting hypothesis could establish a direct link between the tendency to aggregate and the genomic code.

In their paper, the authors have tested the hypothesis on the genomes of eubacteria using a genome-wide approach based on multiple machine learning models. Eubacteria are an interesting set of organisms which have an appreciably high variation in their nucleotide composition with the percentage of CG genetic material ranging from 20% to 70%. The authors classified different eubacterial proteomes in terms of their aggregation propensity and chaperone-dependence. For this purpose, new classifiers had to be developed which were based on carefully curated data. They took account for twenty-four different features among which are sequence patterns, the pseudo amino acid composition of phenylalanine, aspartic and glutamic acid, the distribution of positively charged amino acids, the FoldIndex score and the hydrophobicity. These classifiers seem to be altogether more accurate and robust than previous such parameters.

The authors found that, contrary to what expected from the working hypothesis, which would predict a decrease in protein aggregation with an increase in GC richness, the aggregation propensity of proteomes increases with the GC content and thus the stability of the proteome against aggregation increases with the decrease in GC content. The work also established a direct correlation between GC-poor proteomes and a lower dependence on GroEL. The authors conclude by proposing that a decrease in eubacterial GC content may have been selected in organisms facing proteostasis problems. A way to test the overall results would be through *in vitro *evolution experiments aimed at testing whether adaptation to low GC content provide folding advantage.

The main strengths of this paper is that it addresses an interesting and timely question, finds a novel solution based on a carefully selected set of rules, and provides a clear answer. As such this article represents an excellent and elegant bioinformatics genome-wide study which will almost certainly influence our thinking about protein aggregation and evolution. Some of the weaknesses are the not always easy readability of the text which establishes unclear logical links between concepts.

Another possible criticism could be that, as any *in silico* study, it makes strong assumptions on the sequence features that lead to aggregation and strongly relies on the quality of the classifiers used. Even though the developed classifiers seem to be more robust than previous such parameters, they remain only overall indications which can only allow statistical considerations. It could of course be argued that this is good enough to reach meaningful conclusions in this specific case.

I have read this submission. I believe that I have an appropriate level of expertise to confirm that it is of an acceptable scientific standard.

# Peer review: Referee response for version 1

**DOI:** 10.5256/f1000research.4611.r5273

**Published:** 2014-07-15

**Authors:** Amnon Horovitz

**Affiliations:** 1 Department of Structural Biology, Weizmann Institute of Science, Rehovot, Israel

In this study, the authors describe machine-learning classifiers for predicting aggregation propensities of proteins.  A novel aspect of this work is that the classifiers are based on experimental data obtained from different sources regarding chaperone dependence (GroEL or DnaK) and solubility in chaperone-free systems. The authors then use their machine-learning approach to test for ~1100 eubacterial proteomes whether a low GC content correlates with a greater tendency to aggregate or mis-fold as suggested by earlier studies.  One possibility is that the GC content affects the amino acid composition of proteins that, in turn, affects their folding and aggregation propensities. The authors show in Figure 3 of the paper that amino acid compositions do indeed correlate strongly with GC content (the Figure shows data for all 20 naturally occurring amino acids, although its title suggests otherwise). They then show that aggregation-prone proteins have a higher GC content than soluble proteins. This finding is not really new since it has already been reported (see ref. 21 in the paper) that GroEL substrates tend to have a relatively high GC content.  However, the observation for a large number of genomes that the fraction of aggregation-prone proteins increases with an increasing GC content is novel.  A weakness of the paper is that the authors do not discuss the problems of distinguishing between (i) causation and correlation; and (ii) cause and effect.  In other words, is the correlation between high GC content and an increased tendency to mis-fold due to some unidentified factor(s) that correlates with both GC content and mis-folding or to a direct effect? In addition, it is also possible (at least in principle) that a high GC content reflects selection against mis-folding rather than being one of its ‘causes’.

I have read this submission. I believe that I have an appropriate level of expertise to confirm that it is of an acceptable scientific standard.
